# Comparative efficacy and safety of first-line treatments for advanced non-small cell lung cancer with *ALK*-rearranged: a meta-analysis of clinical trials

**DOI:** 10.1186/s12885-021-08977-0

**Published:** 2021-11-26

**Authors:** Hao-chuan Ma, Yi-hong Liu, Kai-lin Ding, Yu-feng Liu, Wen-jie Zhao, Yan-juan Zhu, Xue-song Chang, Ya-dong Chen, Zhen-zhen Xiao, Ya-ya Yu, Rui Zhou, Hai-bo Zhang

**Affiliations:** 1 Department of Oncology, the Second Affiliated Hospital of Guangzhou University of Chinese Medicine, Guangdong Provincial Hospital of Traditional Chinese Medicine, Guangzhou, 510120 China; 2 The Second Clinical Medical School, Guangzhou University of Chinese Medicine, Guangzhou, 510120 China; 3 Guangdong-Hong Kong-Macau Joint Lab on Chinese Medicine and Immune Disease Research, Guangzhou University of Chinese Medicine, Guangzhou, 510120 China; 4 Guangdong Provincial Key Laboratory, of Clinical Research on Traditional Chinese Medicine Syndrome, Guangzhou, 510120 China; 5 State Key Laboratory of Dampness Syndrome of Chinese Medicine, The Second Affiliated Hospital of Guangzhou University of Chinese Medicine, Guangzhou, 510120 China

**Keywords:** ALK, Lung cancer, First-line treatment, Network meta-analysis

## Abstract

**Background:**

Whereas there are many pharmacological interventions prescribed for patients with advanced anaplastic lymphoma kinase (*ALK*)- rearranged non-small cell lung cancer (NSCLC), comparative data between novel generation ALK-tyrosine kinase inhibitors (TKIs) remain scant. Here, we indirectly compared the efficacy and safety of first-line systemic therapeutic options used for the treatment of *ALK*-rearranged NSCLC.

**Methods:**

We included all phase 2 and 3 randomised controlled trials (RCTs) comparing any two or three treatment options. Eligible studies reported at least one of the following outcomes: progression free survival (PFS), overall survival (OS), objective response rate (ORR), or adverse events of grade 3 or higher (Grade ≥ 3 AEs). Subgroup analysis was conducted according to central nervous system (CNS) metastases.

**Results:**

A total of 9 RCTs consisting of 2484 patients with 8 treatment options were included in the systematic review. Our analysis showed that alectinib (300 mg and 600 mg), brigatinib, lorlatinib and ensartinib yielded the most favorable PFS. Whereas there was no significant OS or ORR difference among the ALK-TKIs. According to Bayesian ranking profiles, lorlatinib, alectinib 600 mg and alectinib 300 mg had the best PFS (63.7%), OS (35.9%) and ORR (37%), respectively. On the other hand, ceritinib showed the highest rate of severe adverse events (60%).

**Conclusion:**

Our analysis indicated that alectinib and lorlatinib might be associated with the best therapeutic efficacy in first-line treatment for major population of advanced NSCLC patients with *ALK*-rearrangement. However, since there is little comparative evidence on the treatment options, there is need for relative trials to fully determine the best treatment options as well as the rapidly evolving treatment landscape.

**Supplementary Information:**

The online version contains supplementary material available at 10.1186/s12885-021-08977-0.

## Introduction

Lung cancer is the second most commonly diagnosed cancer and the leading cause of cancer death globally in 2020, with a 5-year survival rate of only 10 to 20% [1], and non-small cell lung cancer (NSCLC) accounts for about 85% of overall reported cases [2]. The anaplastic lymphoma-kinase (*ALK*)-rearrangements are detectable in approximately 2–7% of patients with NSCLC, especially those who are light/never-smokers and younger patients [3–5].

Currently multiple generation ALK-tyrosine kinase inhibitors (TKIs) have been developed (including crizotinib (first generation); alectinib, ceritinib, brigatinib, and ensartinib (second generation); and lorlatinib (third generation)), and most of these TKIs have been established as standard first line treatments [6, 7]. However, despite an initial response to ALK-TKIs, acquired resistance inevitably develops and the way to overcome it is an open challenge [8]. Furthermore, patients harboring an *ALK* -rearrangements are particularly prone to central nervous system (CNS) metastasis, therefore, the ideal treatment could differ in patients stratified by CNS metastasis [9].

Several randomised controlled trials (RCTs) using only the direct comparison model have been conducted for conclusive evidence about the comparative efficacy and safety of first line treatments for patients with advanced *ALK*-rearranged NSCLC [10–14]. However, they have been unable to address the aforementioned problems, especially the second and third-generation TKIs. To provide additional evidence to guide treatment choices for *ALK* -rearranged NSCLC patients, we conducted a Bayesian network meta-analysis to comprehensively integrate all available direct and indirect evidence with a well-designed and comparative synthesis.

## Methods

To conduct this network meta-analysis, we followed the preferred reporting items for systematic reviews and meta-analyses (PRISMA) extension statement for network meta-analysis (Additional files Table S1). The protocol of this study had been registered in the International Prospective Register of Systematic Reviews (PROSPERO), under the registration number of CRD42020173238 [15, 16].

### Data sources and searches

We searched PubMed, Embase, Cochrane Library, and ClinicalTrials.gov databases to find relevant articles up to 10 Sep 2021 in all languages. Then, to include complete and updated outcomes, abstracts of ongoing RCTs on NSCLC from several of the most important international conferences (American Society of Clinical Oncology, European Society of Medical Oncology, European Cancer Conference, and World Conference on Lung Cancer) from 2016 to 2021 were inspected. We used the following key words:“non-small-cell lung cancer”, “NSCLC”, “non-small-cell lung carcinoma”, “treatment”, “ALK”,“TKIs”, “randomized controlled trial”, and “clinical trial”. The detailed search strategy is presented in Additional files Table S2.

### Study selection

Two independent investigators (MHC and LYF) screened relevant records to identify all the relevant RCTs, and any disagreements were resolved via consensus. We included phase II/III RCTs involving adult patients with histologically or cytologically confirmed advanced (stage III/IV/recurrent) NSCLC with *ALK*- rearrangement, comparing any two or more first line treatments. Studies that compared different doses of one ALK-TKI were also included.

As the current standard of first-line therapy for patients with advanced *ALK* -rearranged NSCLC is treatment with ALK-TKIs, the comparator of this meta-analysis is dominated by ALK-TKIs, and the comparison between chemotherapy were not included in our analyses. Unpublished data and case reports were also excluded from the analysis.

### Data extraction

Two independent investigators (DKL and ZWJ) extracted relevant data parameters. Disagreements were resolved by discussion with the section partner. The following data parameters were extracted: study ID, first author, year of publication, region, RCT design, number of participants in each arm, total number of patients, patients in safety analysis, patient characteristics, interventions, and outcomes. Survival data assessed by an independent review facility were extracted to avoid potential assessment bias by the investigators.

### Risk of bias assessment

The risk of bias of the included original studies was assessed using a modified version of the Cochrane Collaboration’s Risk-of Bias Tool [17]. The following domains were assessed: (1) sequence generation; (2) allocation concealment; (3) blinding of participants, personnel, and outcome assessors; (4) incomplete outcome data; (5) selective outcome reporting; and (6) other potential threats to validity. Each aspect was assigned an assessment index associated with the risk of bias classified as yes, no, or unclear.

### Clinical outcomes

The prespecified outcomes were progression free survival (PFS), overall survival (OS), objective response rate (ORR), and adverse events of grade 3 or higher (Grade ≥ 3 AEs). PFS and OS were analyzed as a survival outcome and reported as Hazard Rate (HR) with an associated 95%CrI. ORR and Grade ≥ 3 AEs were analyzed as a binary outcome and reported as odds ratio (OR) with an associated 95%CrI.

### Statistical analyses

The network meta-analyses were performed with a Bayesian hierarchical random effects model using GeMTC (version 0.14.3) and R (version 3.5.3; Package gemtc) [18, 19]. Model fit was assessed using deviance information criteria (DIC) and random effects standard deviation [20]. A three-chain or four-chain model with non-informative priors was run with an adaptation phase of 40,000 iterations followed by 200,000 model iterations with 20,000 burn-ins for each chain (the thinning interval was 10) [21, 22]. Model convergence was estimated by the Brooks-Gelman-Rubin diagnostic and Potential Scale Reduction Factor (PSRF). In addition, the software can calculate the probability that each intervention is rated as the best (or worst), second, third, etc. Based on these data, we use GraphPad Prism (version 7.0) [23] to showed them in ranking plots.

Subgroup analysis was conducted according to CNS metastases. In order to measure the consistency of the effect size (OR and HR), we performed pairwise meta-analyses to assess heterogeneity between the studies using the Q test and I^2^ statistic in Stata (version 14.0) [24]. Since the number of included studies is less than 10, we did not perform publication bias assessment. Sensitivity analyses were performed to assess the robustness and reliability of the results within each network meta-analysis.

## Results

### Study selection and characteristics

**Fig. 1:**
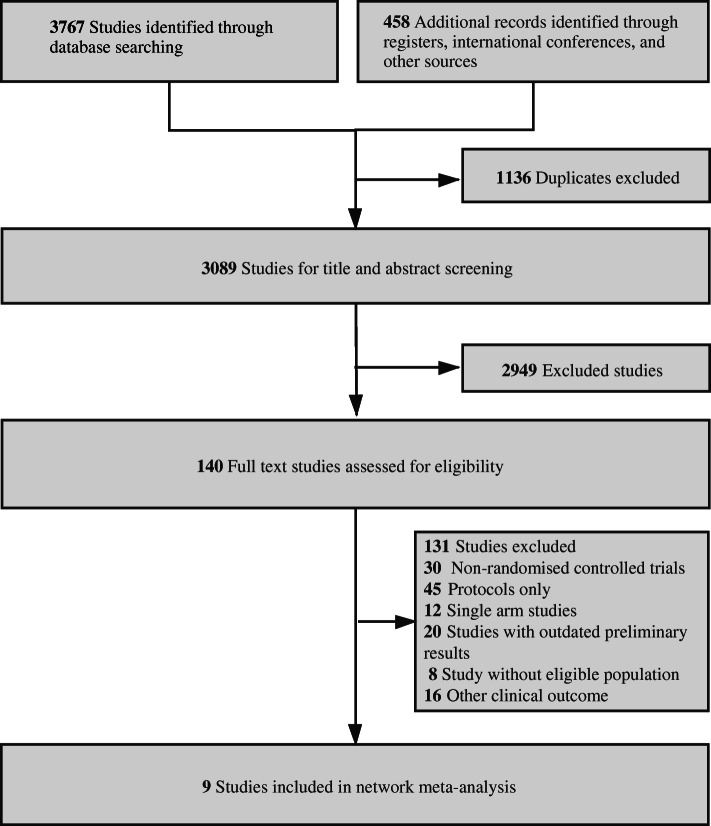
Flowchart of study selection

A total of 4225 records were identified and screened (Fig. 1). Nine RCTs with a total of 2484 patients enrolled to receive 8 different treatments including ALK-TKIs (crizotinib, alectinib 300 mg, alectinib 600 mg, brigatinib, ensartinib, ceritinib and lorlatinib), and chemotherapy were eligible for inclusion [25–34]. The characteristics and results of the included studies are detailed in Table 1-2. Six trials used crizotinib as the control arm while three used chemotherapies. Assumption of transitivity was accepted (Additional files Table S3), and the risk of bias was evaluated (Additional files Fig. S1).

**Table 1:** Characteristics of the eligible studies

Study name	Publication	Year	Phase	Blind	Stage	Sample size (n)		Treatment	
						Experiment	Control	Experiment	Control
ASCEND-4 [12]	Lancet	2017	III	open-label	III, IV	189,187		Ceritinib 750 mg/day	Pemetrexed 500 mg/ m^2^ plus cisplatin 75 mg/m^2^ or carboplatin AUC 5–6
PROFILE 1014 [7, 25]	NEJM, JCO	2014, 2018	III	open-label	III, IV, recurrent	172,171		Crizotinib 250 mg bid	Pemetrexed 500 mg/ m^2^ plus cisplatin 75 mg/m^2^ or carboplatin AUC 5–6
ALEX [10, 26]	NEJM, Ann Oncol	2017, 2020	III	open-label	III, IV	152,151		Alectinib 600 mg bid	Crizotinib 250 mg bid
ALTA-1 L [11, 27]	NEJM, JCO	2018, 2020	III	open-label	III, IV	137,138		Brigatinib 180 mg/day after a 7-day lead-in period of 90 mg/day	Crizotinib 250 mg bid
eXalt3 [13, 28]	WCLC, JAMA Oncology	2020, 2021	III	open-label	III, IV	143,147		Ensartinib 225 mg qd	Crizotinib 250 mg bid
J-ALEX [29–31]	Lancet, Lung cancer, ASCO	2017, 2019, 2021	III	open-label	IIIB, IV, recurrent	103,104		Alectinib 300 mg bid	Crizotinib 250 mg bid
PROFILE 1029 [32]	JTO	2018	III	open-label	III, IV, recurrent	104,103		Crizotinib 250 mg bid	Pemetrexed 500 mg/ m^2^ plus cisplatin 75 mg/m^2^ or carboplatin AUC 5–6
ALESIA [33]	Lancet Respir Med	2019	III	open-label	IIIB, IV	125 62		Alectinib 600 mg bid	Crizotinib 250 mg bid
CROWN [34]	NEJM	2020	III	open-label	III, IV	149,147		Lorlatinib 100 mg qd	Crizotinib 250 mg bid

AUC, Area Under Curve; NEJM, New England Journal Medicine; JCO, Journal of Clinical Oncology; Ann Oncol, Annals of Oncology; WCLC, World conference on lung cancer; ASCO, American Society of Clinical Oncology congress; JTO, Journal of Thoracic Oncology; Lancet Respir Med, The Lancet Respiratory Medicine

**Table 2:** Characteristics of the eligible studies

Study	Smoker (%)		Age (median)		Female (%)		ECOG 0 or 1(%)		Brain metastases (%)		Reported outcomes
	Exp	Con	Exp	Con	Exp	Con	Exp	Con	Exp	Con	
ASCEND-4 [12]	43 35		55 54		54.0 61.0		94 93		31 33		PFS, OS, ORR, Grade ≥ 3 AEs
PROFILE 1014 [7, 25]	39 35		52 54		60.5 63.2		94 95		26 27		PFS, OS, ORR, Grade ≥ 3 AEs
ALEX [10, 26]	40 35		58 54		55.3 57.6		93 93		42 38		PFS, OS, ORR, Grade ≥ 3 AEs
ALTA-1 L [11, 27]	39 46		58 60		50.4 58.7		96 96		29 30		PFS, OS, ORR, Grade ≥ 3 AEs
eXALT3 [13, 28]	40.6 36.1		54 53		49.7 47.6		95.1 95.2		32.9 38.8		PFS, OS, ORR, Grade ≥ 3 AEs
J-ALEX [29–31]	46 41		61 59.5		60 61		98 98		14 28		PFS, OS, ORR, Grade ≥ 3 AEs
PROFILE 1029 [32]	25 30.1		48 50		51.9 58.3		96.2 96.1		20.2 31.1		PFS, OS, ORR, Grade ≥ 3 AEs
ALESIA [33]	33 28		51 49		48.8 45.2		97 98		35 37		PFS, ORR, Grade ≥ 3 AEs
CROWN [34]	46 35		61 56		56.0 62.0		98 94		26 27		PFS, OS, ORR, Grade ≥ 3 AEs

Exp, Experiment; Con, Control; PFS, progression free survival; OS, overall survival; ORR, objective response rate; Grade ≥ 3 AEs, adverse events of grade 3 or higher

### Network meta-analysis for advanced *ALK*-rearranged NSCLC

**Fig. 2:**
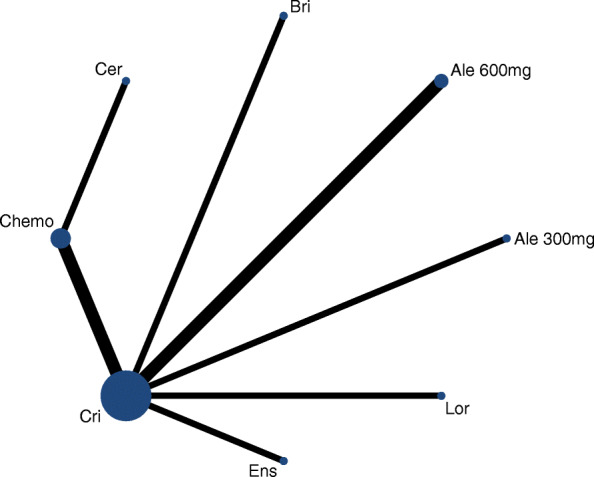
Network plot comparing different treatment outcomes in different groups of patients with advanced *ALK*-rearranged NSCLC

The network meta-analysis included 9 studies for PFS, OS, ORR and Grade ≥ 3 AEs (Fig. 2).

#### Progression free survival

**Fig. 3:**
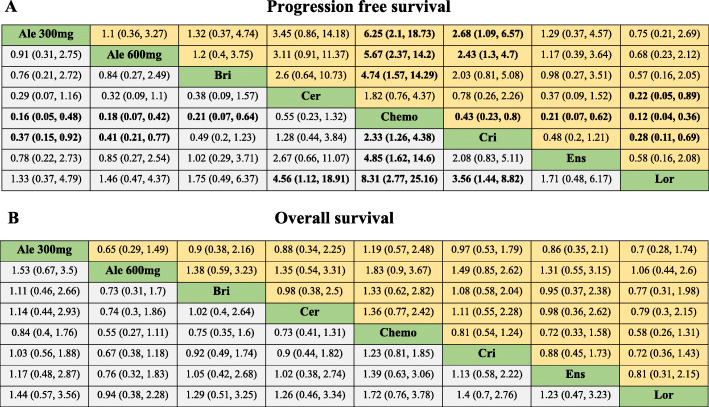
Pooled estimates of the network meta-analysis. (A) Pooled hazard ratios (95% credible intervals) for progression free survival. (B) Pooled odds ratios (95% credible intervals) for overall survival. Data in each cell are hazard or odds ratios (95% credible intervals) for the comparison of row-defining treatment versus column-defining treatment. Hazard ratios less than 1 favour row-defining treatment. Significant results are in bold. Ale 300 mg, alectinib 300 mg; Ale 600 mg, alectinib 600 mg; Bri, brigatinib; Cer, ceritinib; Chemo, chemotherapy; Cri, crizotinib; Ens, ensartini; Lor, lorlatinib

**Fig. 4:**
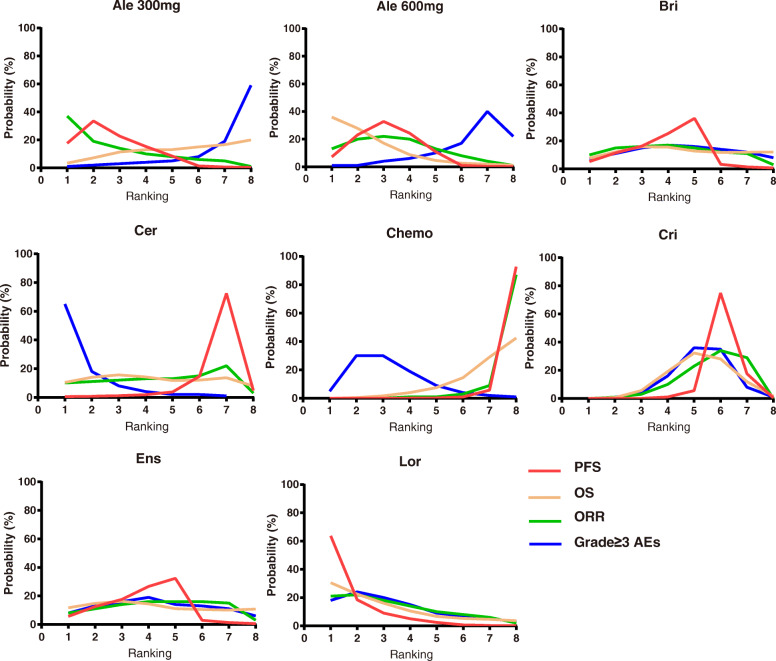
Bayesian ranking profiles of comparable treatments on efficacy for patients with advanced *ALK*-rearranged, non-small cell lung cancer. The profiles indicate the probability of each comparable treatment being ranked from first to last on progression free survival, overall survival, objective response rate, and grade ≥ 3 adverse events. Ale 300 mg, alectinib 300 mg; Ale 600 mg, alectinib 600 mg; Bri, brigatinib; Cer, ceritinib; Chemo, chemotherapy; Cri, crizotinib; Ens, ensartini; Lor, lorlatinib

Lorlatinib and alectinib (300 mg and 600 mg) were significantly superior to ceritinib, crizotinib and chemotherapy (Fig. 3). Lorlatinib yielded superior PFS against ceritinib (HR 0.22, 95%CrI 0.05 to 0.89), crizotinib (HR 0.28, 95%CrI 0.11 to 0.69), and chemotherapy (HR 0.12, 95%CrI 0.04 to 0.36). On the other hand, alectinib 600 mg showed better PFS against ceritinib (HR 0.32, 95%CrI 0.09 to 1.10), crizotinib (HR 0.41, 95%CrI 0.21 to 0.77), or chemotherapy (HR 0.18, 95%CrI 0.07 to 0.42), this result is broadly consistent with the results with that of alectinib 300 mg. Brigatinib and ensartinib yielded results comparable with lorlatinib and alectinib. According to Bayesian ranking profiles, lorlatinib had the highest PFS (63.7%), followed by alectinib 300 mg (17.6%) and alectinib 600 mg (7.2%) (Fig. 4).

#### Overall survival

Due to the short follow-up duration, the OS data of most studies remain immature, which may affect to some extent the outcome of this analysis. Our OS analysis showed that there was no significant difference among the ALK-TKIs or between the ALK-TKIs and chemotherapy. However, alectinib 600 mg was a preferred option for OS (Fig. 3). Alectinib 600 mg versus brigatinib (HR 0.73, 95%CrI 0.31 to 1.7), ceritinib (HR 0.74, 95%CrI 0.3 to 1.86), chemotherapy (HR 0.55, 95%CrI 0.27 to 1.11), crizotinib (HR 0.67, 95%CrI 0.38 to 1.18), ensartinib (HR 0.76, 95%CrI 0.32 to 1.83), lorlatinib (HR 0.94, 95%CrI 0.38 to 2.28), and alectinib 300 mg(HR 0.65, 95%CrI 0.29 to 1.49). According to Bayesian ranking profiles, alectinib 600 mg had the highest probability (35.9%) for better OS, followed by lorlatinib (30.6%) and ensartinib (11.8%) (Fig. 4).

#### Objective response rate

**Fig. 5:**
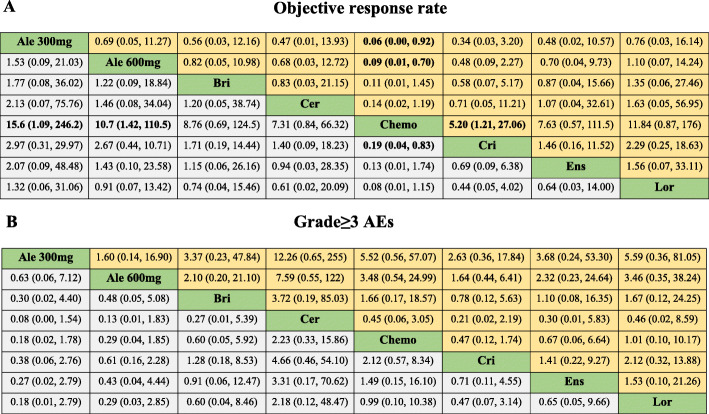
Pooled estimates of the network meta-analysis. (A) Pooled odds ratios (95% credible intervals) for objective response rate. (B) Pooled odds ratios (95% credible intervals) for adverse events of grade 3 or higher. Data in each cell are hazard or odds ratios (95% credible intervals) for the comparison of row-defining treatment versus column-defining treatment. Odds ratios more than 1 favour row-defining treatment. Significant results are in bold. Ale 300 mg, alectinib 300 mg; Ale 600 mg, alectinib 600 mg; Bri, brigatinib; Cer, ceritinib; Chemo, chemotherapy; Cri, crizotinib; Ens, ensartini; Lor, lorlatinib; Grade ≥ 3 AEs, adverse events of grade 3 or higher

Here, whereas there was no significant ORR difference between the ALK-TKIs, the ALK-TKIs were shown to have significantly better ORR compared to chemotherapy (Fig. 5). Comparatively, chemotherapy versus alectinib 300 mg (HR 0.06, 95%CrI 0.00 to 0.92), alectinib 600 mg (HR 0.09, 95%CrI 0.01 to 0.70), ceritinib (HR 0.14, 95%CrI 0.02 to 1.19), brigatinib (HR 0.11, 95%CrI 0.01 to 1.45), crizotinib (HR 0.19, 95%CrI 0.04 to 0.83), ensartinib (HR 0.13, 95%CrI 0.01 to 1.74), and lorlatinib (HR 0.08, 95%CrI 0.01 to 1.15). According to Bayesian ranking profiles, alectinib 300 mg had the highest probability (37%) for better ORR followed by lorlatinib (21%), and alectinib 600 mg (13%) (Fig. 4).

#### Adverse events

For adverse events of grade 3 or higher, different toxicity spectrums were interrogated for individual ALK-TKIs. There was no significant difference between the various treatment options in Grade ≥ 3 AEs (Fig. 5). According to Bayesian ranking profiles, ceritinib was most likely to be associated with the highest (60%) cause of in Grade ≥ 3 AEs, followed by lorlatinib (18%). On the other hand, alectinib 300 mg had the highest probability (59%) of being the safest intervention, followed by alectinib 600 mg (22%) (Fig. 4).

In case of mild AEs (Grade1 and Grade2 AEs), no significant differences among the various treatment options. Based on Bayesian ranking profiles, alectinib 300 mg most likely leads to mild AEs (73%), followed by alectinib 600 mg (17%)(Additional files Fig. S2). This result is negatively associated with Grade ≥ 3 AEs.

Among all grades of adverse events, crizotinib was associated with the worst safety, with a probability of causing 62% hepatic injuries (ALT increase), followed by ensartinib (17%), brigatinib (9%), and lorlatinib (6%). As for renal injury (creatinine increased), the worst safety drugs were brigatinib (68%), alectinib 600 mg (21%) (Additional files Table S4 and Fig. S3).

In addition, crizotinib was associated with 37% probability of causing nausea while alectinib did not cause any forms of nausea. Crizotinib was associated with the worst episodes of diarrhea (probability = 67%) but not alectinib 300 mg. Vomiting events were associated with ensartinib (90%), followed by ceritinib (5%), brigatinib (3%), and chemotherapy (2%) (Additional files Fig. S3).

In terms of bone marrow suppression, chemotherapy was the highest cause of leukopenia (probability = 88%) while alectinib 600 mg had a 2% chance of causing leukopenia. Similarly, chemotherapy had 40% chances of causing anemia, but not crizotinib. The worst safety ranking of neutropenia from high to low was chemotherapy (77%), crizotinib (13%), ceritinib (5%), alectinib 600 mg (2%), and brigatinib (2%) (Additional files Fig. S3).

### Subgroup analysis based on CNS status

**Fig. 6:**
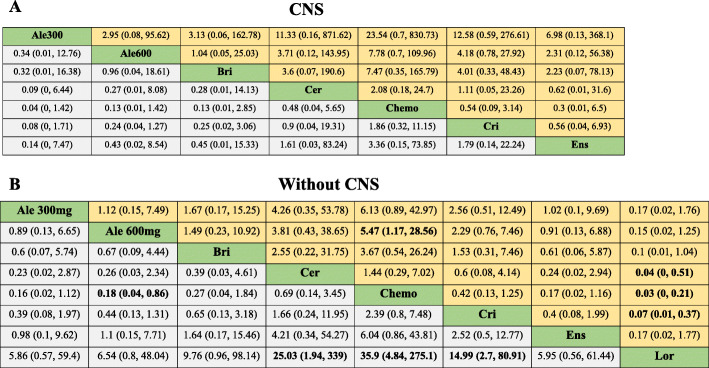
Pooled estimates of the network meta-analysis. (A) Pooled hazard ratios (95% credible intervals) for patients with the baseline CNS metastases. (B) Pooled hazard ratios (95% credible intervals) for patients without baseline CNS metastases. Data in each cell are hazard (95% credible intervals) for the comparison of row-defining treatment versus column-defining treatment. Hazard ratios less than 1 favour row-defining treatment. Significant results are in bold. Ale 300 mg, alectinib 300 mg; Ale 600 mg, alectinib 600 mg; Bri, brigatinib; Cer, ceritinib; Chemo, chemotherapy; Cri, crizotinib; Ens, ensartini; Lor, lorlatinib; CNS, central nervous system

Nearly, all 9 studies included CNS metastases as a stratification factor in randomization except for PROFILE 1029 and J-ALEX. However, only PFS network meta-analysis could be conducted involved 9 studies for the patients without CNS metastases at baseline and 8 studies with CNS metastases. In patients with the baseline CNS metastases except CROWN study, alectinib 300 mg and alectinib 600 mg were more likely to be the best therapeutic options. Our comparative analysis showed alectinib 300 mg versus alectinib 600 mg (HR 0.34, 95%CrI 0.01 to 12.76), brigatinib (HR 0.32, 95%CrI 0.01 to 16.38), ceritinib (HR 0.09, 95%CrI 0.00 to 6.44), chemotherapy (HR 0.04, 95%CrI 0.00 to 1.42), crizotinib (HR 0.08, 95%CrI 0.00 to 1.71),and ensartinib (HR 0.14, 95%CrI 0.00 to 7.74). In addition, alectinib 600 mg versus brigatinib (HR 0.96, 95%CrI 0.04 to 18.61), ceritinib (HR 0.27, 95%CrI 0.01 to 8.08), chemotherapy (HR 0.13, 95%CrI 0.01 to 1.42), crizotinib (HR 0.24, 95%CrI 0.04 to 1.27), and ensartinib (HR 0.43, 95%CrI 0.02 to 8.54). (Fig. 6). According to Bayesian ranking profiles, alectinib 300 mg had the highest probability (63.9%) for better PFS followed by brigatinib (15.1%) (Additional files Fig. S4).

As for the patients without baseline CNS metastases, lorlatinib was the most preferred treatment option. Lorlatinib versus alectinib 300 mg (HR 0.17, 95%CrI 0.02 to 1.76), alectinib 600 mg (HR 0.15, 95%CrI 0.02 to 1.25), brigatinib (HR 0.1, 95%CrI 0.01 to 1.04), ceritinib (HR 0.04, 95%CrI 0.00 to 0.51), chemotherapy (HR 0.03, 95%CrI 0.00 to 0.21), crizotinib (HR 0.07, 95%CrI 0.01 to 0.37), and ensartinib (HR 0.17, 95%CrI 0.02 to 1.77) (Fig. 6). According to Bayesian ranking profiles, lorlatinib had the highest probability (91%) for PFS, followed by alectinib 300 mg (2.74%) (Additional files Fig. S5).

### Assessment of heterogeneity and inconsistency

Forest plots for four feasible pairwise comparisons showed low heterogeneity. However, there was high (75.0%) heterogeneity between alectinib 600 mg and crizotinib, for Grade ≥ 3 AEs (Additional files Fig. S6 and Fig. S7).

## Sensitivity analysis

The sensitive analysis was performed by excluding the J-ALEX study, the results were stable and were similar to main analysis (Additional files Fig. S8 and Fig. S9).

## Discussion

### Principal findings

In this systematic review and network meta-analysis, we comprehensively summarized the comparative effectiveness and safety of multiple first line treatment options for patients with advanced *ALK*-rearranged NSCLC. Our data showed that alectinib (300 mg and 600 mg) and lorlatinib had favorable effectiveness with tolerable adverse effects. On the other hand, toxicity profiles showed that ceritinib resulted in the highest rate of severe adverse events.

The efficacy of crizotinib as the first-line treatment for patients with advanced *ALK*-rearranged NSCLC was proved in PROFILE 1014 [6, 25], however, novel-generation ALK-TKIs in the first-line setting have shown improved PFS versus crizotinib [10, 11, 13, 26–31, 33, 34]. This might be because of its poor brain penetrance and lower intracranial response [35]. Previous studies have shown that crizotinib, a P-glycoprotein (P-gp) substrate, is easily excreted from the blood-brain barrier (BBB) by active efflux with a cerebrospinal fluid concentration of only 0.26% of plasma concentration, compromising the attainment of effective drug concentrations at the CNS, moreover, it has a very low cerebrospinal fluid (CSF) penetration of 0·26% and CSF:IC50 of 0·03 [36, 37]. The second generation ALK-TKIs are non-P-gp substrates, have lower binding to transport proteins which prevent the drug from being excreted from the brain. Therefore, while its BBB penetration rate is significantly increased, with a penetration rate of 63 to 94%, the probability of CNS progression is reduced [38, 39]. Related basic experiments also demonstrated that the CSF penetration and CSF:IC50 value of the second generation ALK-TKIs were significantly higher than the first generation ALK-TKIs, with an improved drug specificity and efficacy [40–42].

There is a lack of head-to-head comparative evidence between the novel-generation ALK-TIKs. Consistent with our findings, previous studies have associated alectinib and lorlatinib with favorable effectiveness [10, 26, 29–31, 33, 34]. The median PFS of alectinib was more than 30 months in two large phase 3 RCTs (ALEX and J-ALEX) [26, 30], while that of brigatinib and ceritinib was 24 months and 16.6 months respectively [12, 27]. AS for OS, median OS was not reached with alectinib 600 mg versus 57.4 months with crizotinib (HR 0.67, 95%CrI 0.46–0.98) in the ALEX study [26]. Although OS data were immature, trends toward improvement will likely sustain and the detail reasons could be concluded as follows. First, the updated data from the ALEX study showed that the 5-year OS rate of alectinib 600 mg exceeded 62.5% while 45.5% for crizotinib, the difference is clinically meaningful [26]. Second, a series of first-line treatment studies on EGFR-TKIs have shown that long-term PFS benefits may be translated into OS, when the OS data is immature [43, 44]. Therefore, PFS is an important indicator in predicting overall survival. Lastly, alectinib may increase overall survival because of the better control of the intracranial lesions. In contrast, the final OS analysis of J-ALEX indicated that OS HR was 1.03 (95%CI 0.67–1.58) and 5-year OS rate in the alectinib 300 mg and crizotinib arm were 60.85 and 64.11%, respectively [31]. Alectinib 300 mg did not demonstrate a significant overall survival benefit versus crizotinib. The acceptance of cross-over in clinical trial might be causable for this difference between ALEX and J-ALEX. The data showed that 83.7% of the patients in the crizotinib arm received alectinib 300 mg as subsequent therapy when follow-up was 42.4 months, and 78.8% in the final OS analysis. On the other hand, ALEX study and J-ALEX study used different doses of alectinib, this may be one possible reason for this difference in OS, but this needs to be further confirmed.

In addition, alectinib has a broad-spectrum of safety medications. Our results reveal no obvious differences in terms of severe AEs rates and mild AEs rates in 600 mg and 300 mg. Similarly, Gainor et al. increased the dose of alectinib to 900 mg in a case report, and did not observe any drug-related adverse reactions, but mild constipation [45]. This increased drug dosages led to an increase in the intracranial drug concentration of alectinib, while the other second generation ALK-TIKs had limited increase of intracranial drug concentration due to adverse reaction. Thus, this explains why alectinib has may have yielded better control of CNS metastases, as a first-line treatment.

Similarly, lorlatinib provides improved control of CNS disease, 71% of the lorlatinib arm patients had an intracranial complete response in CROWN study [34]. Zou et al. also showed that lorlatinib induced superior regression of intracranial *EML4-ALK* tumors and prolonged survival in mice, compared with crizotinib. Moreover, the enhanced activity of lorlatinib on brain metastasis model was attributed to its ability to cross the BBB [46, 47]. Besides, lorlatinib remain the antitumor activity for all known single ALK resistance mutations [48, 49]. These factors might be the basis for the marked efficacy of lorlatinib as first-line therapy.

Regarding safety, our studies demonstrate that ceritinib 750 mg are expected to be at the highest risk of developing severe adverse effects. In the ASCEND-1 and ASCEND-4 study [12, 50], administration of ceritinib at a dose of 750 mg led to 56–80% grade 1–2 gastrointestinal AEs, and about 5% grade 3 gastrointestinal AEs, which was consistent with our finding. However, we only incorporated ASCEND-4 study that was 750 mg, analysis of other doses was lacking. In the ASCEND-8 study, the researchers adjusted the ceritinib dosage to explored whether it can effectively reduce the gastrointestinal side effects [51]. They evaluated the efficacy and safety of ceretinib 450 mg/600 mg compared with 750 mg in *ALK*-positive advanced NSCLC patients. The results showed that compared with the 750-mg fasted dose, the frequency of dose reduction and withdrawal of ceritinib 450-mg was significantly lower, the median dose intensity was higher, and the frequency and severity of gastrointestinal adverse reactions are significantly reduced. These ASCEND-8 results were similar to the data from real-world studies (RWS) [52].

### Strengths and limitations

We conducted a meta-analysis to compare the safety and adverse events of all ALK-TKIs approved by FDA. The results showed that alectinib was the safest second-generation TKI. Besides, SAE occurred in 40% of the patients treated with ceritinib and brigatinib [53]. Another Network meta-analysis on WCLC2019 indirectly compared the efficacy of the second-generation ALK inhibitors, and showed that ceritinib (450 mg taken with food) has similar effect as alectinib [54]. Compared with other previous meta-analyses investigating treatments for patients with advanced *ALK*-rearranged NSCLC, our network meta-analysis only included RCTs, reducing the impact of heterogeneity by study design.

However, most of the trials included in our analysis had immature OS data, without median OS. The limited data on the OS suggest that it might cause heterogeneity when taken as an endpoint to evaluate each personal treatment’s realistic effect. Besides, most of the included studies are mainly clinical trials, posing potential publication and selection bias. Thirdly, only the data on PFS was included in the subgroup analysis, while the data on AEs, ORR, and OS was lacking. As a final note, patients were not stratified according to factors like race, which might modify treatment benefits, appropriate dose and efficacy of ALK-TKIs in the Asian population may differ from the Western population, Subsequent studies should investigate the relative treatment efficacy according to these clinical characteristics.

### Implications

By synthesizing all the evidence in the RCTs, this review provides clinicians a reference source to evaluate strengths and weaknesses associated with all the promising therapeutic options. Due to its promising effectiveness and safety, alectinib and lorlatinib were recommended as the first-line treatment option for advanced NSCLC patients with *ALK*-rearrangement. Therefore, these findings can help answer questions on whether there were differences in the efficacy and safety of different doses of alectinib compared with the other ALK-TKIs, in controlling CNS metastasis. Besides, the review shows whether ensartinib and brigatinib can challenge the use of alectinib as the first-line treatment.

We, however, recommend that there should be more head-to-head RCTs between the second- generation and third-generation ALK-TKIs. After ALK-TKI drug resistance, it is necessary to interrogate how to carry out reasonable sequential treatment, optimize the whole medication arrangement and achieve the longest survival time. Moreover, it is also necessary to continue exploring the mechanism of resistance to provide guidance for clinical applications in basic research.

## Conclusions

These results indicated that alectinib and lorlatinib might be associated with the best therapeutic efficacy in first-line treatment for major population of advanced NSCLC patients with *ALK*-rearrangement.

## Supplementary Information

**Additional file 1.**

## Data Availability

All data generated or analyzed during this study are included in this published article [and its supplementary information files].

## Abbreviations

NSCLC: Non-small cell lung cancer
ALK: Anaplastic lymphoma kinase
TKIs: Tyrosine kinase inhibitors
ORR: Objective response rate
PFS: Progression free survival
CNS: Central nervous system
RCTs: Randomised controlled trials
PRISMA: Preferred Reporting Items for Systematic Reviews and Meta-Analyses
PROSPERO: Prospective Register of Systematic Reviews
OS: Overall survival
Grade ≥ 3 AEs: Adverse events of grade 3 or higher
HR: Hazard Rate
OR: Odds ratio
DIC: Deviance information criteria
PSRF: Potential Scale Reduction Factor
P-gp: P-glycoprotein
BBB: Blood-brain barrier
CSF: Cerebrospinal fluid
RWS: Real-world studies
