# Supplementary material for: Comparative efficacy and safety of first-line treatments for advanced non-small cell lung cancer with ALK-rearranged: a meta-analysis of clinical trials
Source: BMC Cancer. 2021 Nov 26;21:1278. doi: 10.1186/s12885-021-08977-0 (PMC8620528; doi:10.1186/s12885-021-08977-0)
Supplement: Supplementary file 1 — Additional file 1. [file 12885_2021_8977_MOESM1_ESM.docx]

**Comparative efficacy and safety of first-line treatments for advanced non-small cell lung cancer with ALK-rearranged: A meta-analysis of clinical trials**

**Additional files**

**Table S1 Checklist of the PRISMA extension for network meta-analysis**

| **Section/Topic** | **Item #** | **Checklist Item** | **Reported on Page #** |
| --- | --- | --- | --- |
| **TITLE** |  |  |  |
| Title | 1 | Identify the report as a systematic review incorporating a network meta-analysis (or related form of meta-analysis). | 1 |
| **ABSTRACT** |  |  |  |
| Structured summary | 2 | Provide a structured summary including, as applicable:  **Background:** main objectives  **Methods:** data sources; study eligibility criteria, participants, and interventions; study appraisal; and synthesis methods, such as network meta-analysis.  **Results:** number of studies and participants identified; summary estimates with corresponding confidence/credible intervals; treatment rankings may also be discussed. Authors may choose to summarize pairwise comparisons against a chosen treatment included in their analyses for brevity.  **Discussion/Conclusions:** limitations; conclusions and implications of findings.  **Other:** primary source of funding; systematic review registration number with registry name. | 3-4 |
| **INTRODUCTION** |  |  |  |
| Rationale | 3 | Describe the rationale for the review in the context of what is already known, including mention of why a network meta-analysis has been conducted. | 3-4 |
| Objectives | 4 | Provide an explicit statement of questions being addressed, with reference to participants, interventions, comparisons, outcomes, and study design (PICOS). | 3-4 |
| **METHODS** |  |  |  |
| Protocol and registration | 5 | Indicate whether a review protocol exists and if and where it can be accessed (e.g., Web address); and, if available, provide registration information, including registration number. | 6 |
| Eligibility criteria | 6 | Specify study characteristics (e.g., PICOS, length of follow-up) and report characteristics (e.g., years considered, language, publication status) used as criteria for eligibility, giving rationale. Clearly describe eligible treatments included in the treatment network, and note whether any have been clustered or merged into the same node (with justification). | 6-7 |
| Information sources | 7 | Describe all information sources (e.g., databases with dates of coverage, contact with study authors to identify additional studies) in the search and date last searched. | 6 |
| Search | 8 | Present full electronic search strategy for at least one database, including any limits used, such that it could be repeated. | Additional files Table S2 |
| Study selection | 9 | State the process for selecting studies (i.e., screening, eligibility, included in systematic review, and, if applicable, included in the meta-analysis). |  |
| Data collection process | 10 | Describe method of data extraction from reports (e.g., piloted forms, independently, in duplicate) and any processes for obtaining and confirming data from investigators. | 7 |
| Data items | 11 | List and define all variables for which data were sought (e.g., PICOS, funding sources) and any assumptions and simplifications made. | 7 |
| Geometry of the network | S1 | Describe methods used to explore the geometry of the treatment network under study and potential biases related to it. This should include how the evidence base has been graphically summarized for presentation, and what characteristics were compiled and used to describe the evidence base to readers. | 8-9 |
| Risk of bias within individual studies | 12 | Describe methods used for assessing risk of bias of individual studies (including specification of whether this was done at the study or outcome level), and how this information is to be used in any data synthesis. | 7-8 |
| Summary measures | 13 | State the principal summary measures (e.g., risk ratio, difference in means). Also describe the use of additional summary measures assessed, such as treatment rankings and surface under the cumulative ranking curve (SUCRA) values, as well as modified approaches used to present summary findings from meta-analyses. | 7-9 |
| Planned methods of analysis | 14 | Describe the methods of handling data and combining results of studies for each network meta-analysis. This should include, but not be limited to:   - Handling of multi-arm trials; - Selection of variance structure; - Selection of prior distributions in Bayesian analyses; and - Assessment of model fit. | 7-9 |
| Assessment of Inconsistency | S2 | Describe the statistical methods used to evaluate the agreement of direct and indirect evidence in the treatment network(s) studied. Describe efforts taken to address its presence when found. | 7-9 |
| Risk of bias across studies | 15 | Specify any assessment of risk of bias that may affect the cumulative evidence (e.g., publication bias, selective reporting within studies). | 7-9 |
| Additional analyses | 16 | Describe methods of additional analyses if done, indicating which were pre-specified. This may include, but not be limited to, the following:   - Sensitivity or subgroup analyses; - Meta-regression analyses; - Alternative formulations of the treatment network; and - Use of alternative prior distributions for Bayesian analyses (if applicable). | 7-9 |
| **RESULTS†** |  |  |  |
| Study selection | 17 | Give numbers of studies screened, assessed for eligibility, and included in the review, with reasons for exclusions at each stage, ideally with a flow diagram. | 9-10 |
| Presentation of network structure | S3 | Provide a network graph of the included studies to enable visualization of the geometry of the treatment network. | Figure2 |
| Summary of network geometry | S4 | Provide a brief overview of characteristics of the treatment network. This may include commentary on the abundance of trials and randomized patients for the different interventions and pairwise comparisons in the network, gaps of evidence in the treatment network, and potential biases reflected by the network structure. | 9-10 |
| Study characteristics | 18 | For each study, present characteristics for which data were extracted (e.g., study size, PICOS, follow-up period) and provide the citations. | 9-10 |
| Risk of bias within studies | 19 | Present data on risk of bias of each study and, if available, any outcome level assessment. | 9-10 |
| Results of individual studies | 20 | For all outcomes considered (benefits or harms), present, for each study: 1) simple summary data for each intervention group, and 2) effect estimates and confidence intervals. Modified approaches may be needed to deal with information from larger networks. | 10-13 |
| Synthesis of results | 21 | Present results of each meta-analysis done, including confidence/credible intervals. In larger networks, authors may focus on comparisons versus a particular comparator (e.g. placebo or standard care), with full findings presented in an appendix. League tables and forest plots may be considered to summarize pairwise comparisons. If additional summary measures were explored (such as treatment rankings), these should also be presented. | 10-13 |
| Exploration for inconsistency | S5 | Describe results from investigations of inconsistency. This may include such information as measures of model fit to compare consistency and inconsistency models, P values from statistical tests, or summary of inconsistency estimates from different parts of the treatment network. | 10-13 |
| Risk of bias across studies | 22 | Present results of any assessment of risk of bias across studies for the evidence base being studied. | 15 |
| Results of additional analyses | 23 | Give results of additional analyses, if done (e.g., sensitivity or subgroup analyses, meta-regression analyses, alternative network geometries studied, alternative choice of prior distributions for Bayesian analyses, and so forth). | 13-15 |
| **DISCUSSION** |  |  |  |
| Summary of evidence | 24 | Summarize the main findings, including the strength of evidence for each main outcome; consider their relevance to key groups (e.g., healthcare providers, users, and policy-makers). | 15-20 |
| Limitations | 25 | Discuss limitations at study and outcome level (e.g., risk of bias), and at review level (e.g., incomplete retrieval of identified research, reporting bias). Comment on the validity of the assumptions, such as transitivity and consistency. Comment on any concerns regarding network geometry (e.g., avoidance of certain comparisons). | 20-21 |
| **Conclusions** | 26 | Provide a general interpretation of the results in the context of other evidence, and implications for future research. | 22 |
| **FUNDING** |  |  |  |
| Funding | 27 | Describe sources of funding for the systematic review and other support (e.g., supply of data); role of funders for the systematic review. This should also include information regarding whether funding has been received from manufacturers of treatments in the network and/or whether some of the authors are content experts with professional conflicts of interest that could affect use of treatments in the network. | 23-24 |

PRISMA, Preferred Reporting Items for Systematic Reviews and Meta-Analysis; PICOS, population, intervention, comparators, outcomes, study design. Text in italics indicates wording specific to reporting of network meta-analyses that has been added to guidance from the PRISMA statement.

**Table S2** **Literature search criteria**

| Search ((((("Carcinoma, Non-Small-Cell Lung"[Mesh]) OR (((((((((non-small-cell lung cancer[Title/Abstract]) OR NSCLC[Title/Abstract]) OR non-small cell lung cancer[Title/Abstract]) OR non-small-cell lung cancer[Title/Abstract]) OR non-small cell lung cancer[Title/Abstract]) OR non-small-cell lung carcinoma[Title/Abstract]) OR non-small cell lung carcinoma[Title/Abstract]) OR non-small-cell lung carcinoma[Title/Abstract]) OR non-small cell lung carcinoma[Title/Abstract]))) AND ((((((((((((((((((((treatment[Title/Abstract]) OR therapy[Title/Abstract]) OR tyrosine kinase inhibitor[Title/Abstract]) OR TKI[Title/Abstract]) OR alectinib[Title/Abstract]) OR brigatinib[Title/Abstract]) OR ceritinib[Title/Abstract]) OR crizotinib[Title/Abstract]) OR lorlatinib[Title/Abstract]) OR bevacizumab[Title/Abstract]) OR Avastin[Title/Abstract]) OR chemotherapy[Title/Abstract]) OR first-line[Title/Abstract]) OR first line[Title/Abstract]) OR treatment-naïve[Title/Abstract]) OR untreated[Title/Abstract]) OR ALK tyrosine kinase receptor[Title/Abstract]) OR CD246 Antigen[Title/Abstract])) AND (("Anaplastic Lymphoma Kinase"[Mesh]) OR ALK[Title/Abstract]))) AND ((((((compare[Title/Abstract]) OR comparison[Title/Abstract]) OR comparative[Title/Abstract]) OR comparing[Title/Abstract]) OR versus[Title/Abstract]) OR vs[Title/Abstract])) AND ((randomized controlled trial[Publication Type] OR randomized[Title/Abstract] OR placebo[Title/Abstract] OR Clinical Trial [Publication Type] OR Treatment Outcome[Mesh] OR Clinical Study [Publication Type])) |
| --- |

**Table S3 Assessment of transitivity**

| **Characteristic** | **Intervention arm** | **Control arm** |
| --- | --- | --- |
| Sample size | 0.741 | 0.922 |
| Female ratio | 0.148 | 0.222 |
| Smoking status | —— | —— |
| Never | 0.263 | 0.255 |
| Current | 0.274 | 0.233 |
| Former | 0.256 | 0.244 |
| Age | 0.332 | 0.401 |

The above characteristics have been evaluated in all trials included in the network. All of the comparisons had similar median age (left) and other main characteristics with P value over 0.05 (right).

**Table S4 Relative toxicity of treatments on nine commonly reported specific adverse events for ALK-TKIs(Pooled odds ratios for each available comparison on each specific adverse event (any grade)).**

|  | **ALT increase** | **Diarrhea** | **Nausea** | **Vomiting** | **Neutropenia** | **Rash** | **Anemia** | **Leukopenia** | **Creatinine increased** |
| --- | --- | --- | --- | --- | --- | --- | --- | --- | --- |
| **Comparison** | **VS Alectinib300mg** | | | | | | | | |
| Alectinib600mg | 2.06 | 3.57 | 3.46 | 1.31 | 1.66 | 2.32 | —— | —— | 1.58 |
| Brigatinib | 2.09 | 18.63 | 6.09 | 3.77 | 1.01 | 6.44 | —— | —— | 2.87 |
| Crizotinib | 4.29 | 22.77 | 21.65 | 8.79 | 12.83 | 1.11 | —— | —— | 0.82 |
| chemotherapy | —— | 1.60 | 20.32 | 6.79 | 23.77 | 1.00 | —— | —— | —— |
| Ceritinib | —— | —— | —— | 24.06 | 4.07 | —— | —— | —— | —— |
| Lorlatinib | 1.76 | 5.50 | 3.40 | 2.51 | —— | —— | —— | —— | —— |
| Ensartinib | 2.23 | —— | 13.90 | 233 | —— | 536 | —— | —— | —— |
| **Comparison** | **VS Alectinib600mg** | | | | | | | | |
| Brigatinib | 1.02 | 5.10 | 1.76 | 2.93 | 0.60 | 2.71 | —— | —— | 1.82 |
| Crizotinib | 2.07 | 6.37 | 6.23 | 11.28 | 7.92 | 0.48 | 0.19 | 6.74 | 0.51 |
| chemotherapy | —— | 0.46 | 5.84 | 5.20 | 14.55 | 0.43 | 1.42 | 15.35 | —— |
| Ceritinib | —— | —— | —— | 18.38 | 2.43 | —— | 0.45 | 2.66 | —— |
| Lorlatinib | 0.84 | 1.60 | 0.97 | 1.96 | —— | —— | 0.56 | —— | —— |
| Ensartinib | 1.08 | —— | 3.92 | 177 | —— | 207 | 0.07 | —— | —— |
| **Comparison** | **VS Brigatinib** | | | | | | | | |
| Crizotinib | 2.06 | 1.27 | 3.55 | 2.95 | 13.02 | 0.17 | —— | —— | 0.28 |
| chemotherapy | —— | 0.09 | 3.34 | 1.75 | 24.16 | 0.16 | —— | —— | —— |
| Ceritinib | —— | —— | —— | 6.10 | 4.05 | —— | —— | —— | —— |
| Lorlatinib | 0.83 | 0.31 | 0.55 | 0.68 | —— | —— | —— | —— | —— |
| Ensartinib | 1.04 | —— | 2.26 | 577 | —— | 818 | —— | —— | —— |
| **Comparison** | **VS Crizotinib** | | | | | | | | |
| chemotherapy | —— | 0.07 | 0.94 | 0.6 | 1.86 | 0.91 | 7.57 | 2.34 | —— |
| Ceritinib | —— | —— | —— | 2.10 | 0.31 | —— | 2.38 | 0.39 | —— |
| Lorlatinib | 0.40 | 0.25 | 0.16 | 0.23 | —— | —— | 2.92 | —— | —— |
| Ensartinib | 0.51 | —— | 0.46 | 189 | —— | 456 | 0.41 | —— | —— |
| **Comparison** | **VS Chemotherapy** | | | | | | | | |
| Ceritinib | —— | —— | —— | 3.48 | 0.17 | —— | 0.32 | 0.17 | —— |
| Lorlatinib | —— | 3.43 | 0.17 | 0.38 | —— | —— | 0.39 | —— | —— |
| Ensartinib | —— | —— | 0.67 | 133 | —— | 525 | 0.05 | —— | —— |
| **Comparison** | **VS Ceritinib** | | | | | | | | |
| Lorlatinib | —— | —— | —— | 0.11 | —— | —— | 1.24 | —— | —— |
| Ensartinib | —— | —— | —— | 138 | —— | —— | 0.17 | —— | —— |
| **Comparison** | **VS Lorlatinib** | | | | | | | | |
| Ensartinib | 1.28 | —— | 4.05 | 908 | —— | —— | 0.13 | —— | —— |

Pooled odds ratios for each available comparison on each specific adverse event (any grade). Significant values are in bold and colored in yellow.


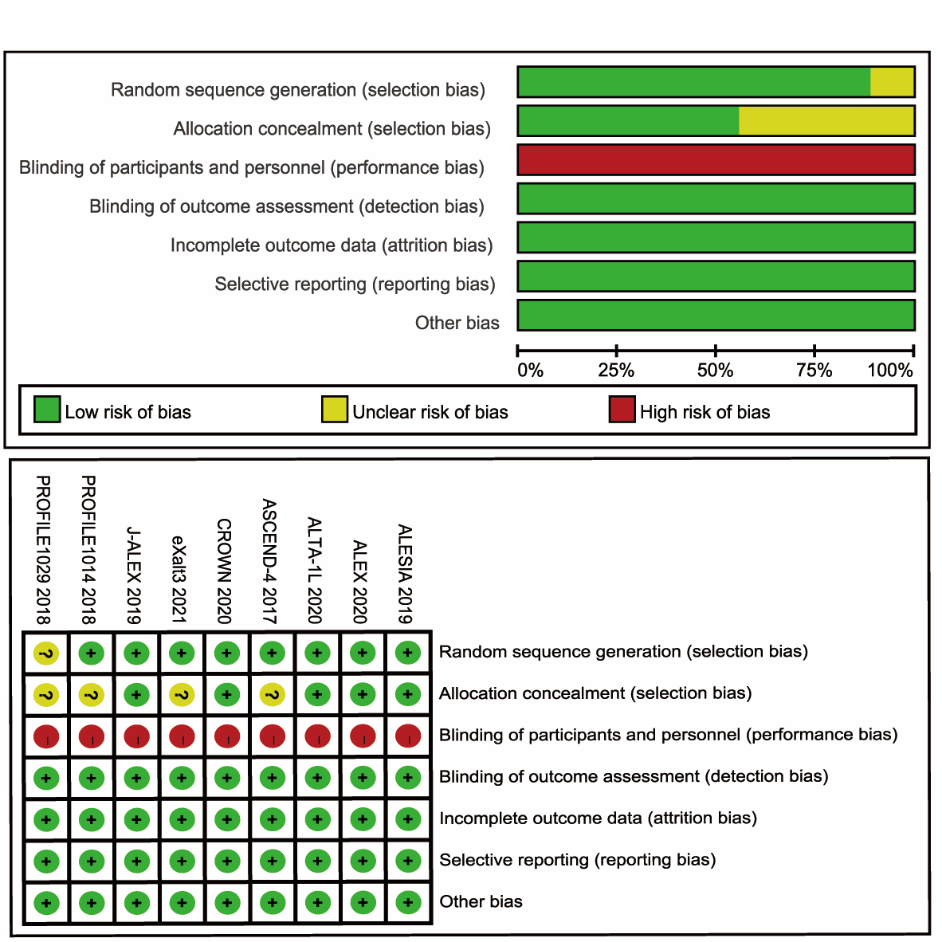


**Figure S1 Summary of results from assessment of studies using the Cochrane risk of bias tool.**


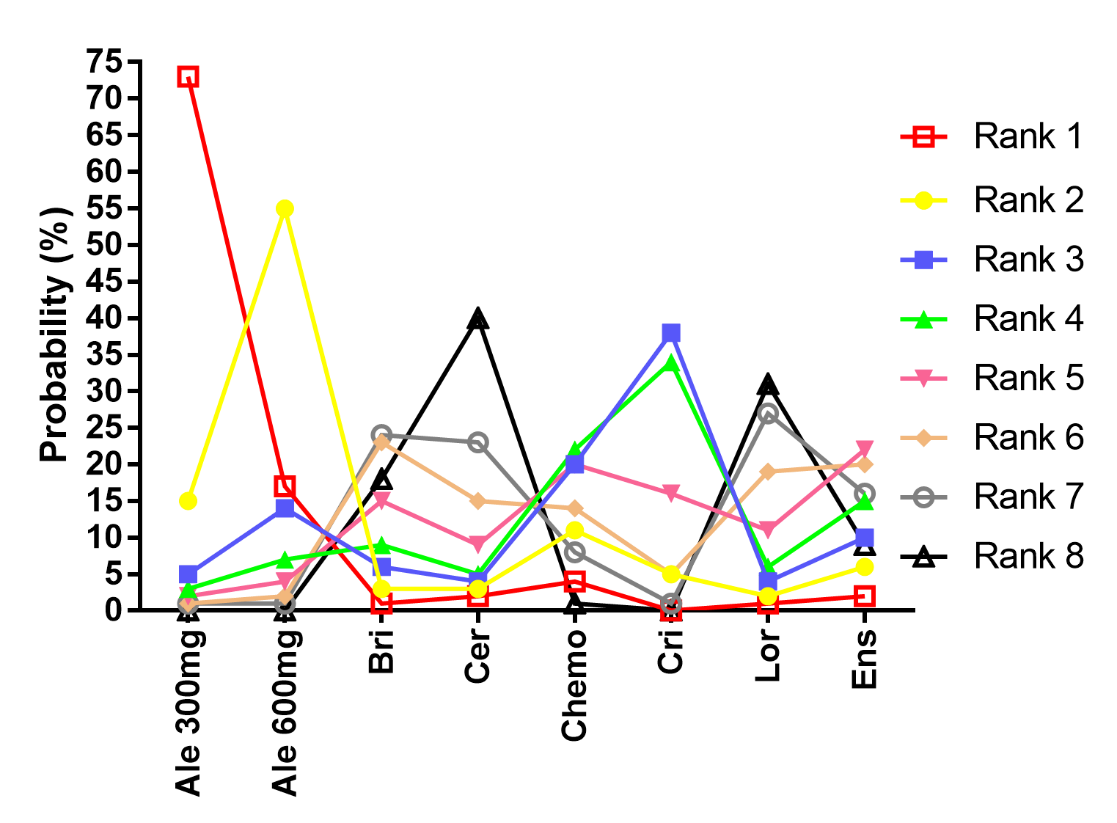


Figure S2 Ranking curves indicating the probability of each comparable treatment on mild adverse events. Ale 300mg, alectinib 300mg; Ale 600mg, alectinib 600mg; Bri, brigatinib; Cer, ceritinib; Chemo, chemotherapy; Cri, crizotinib; Ens, ensartini; Lor, lorlatinib.


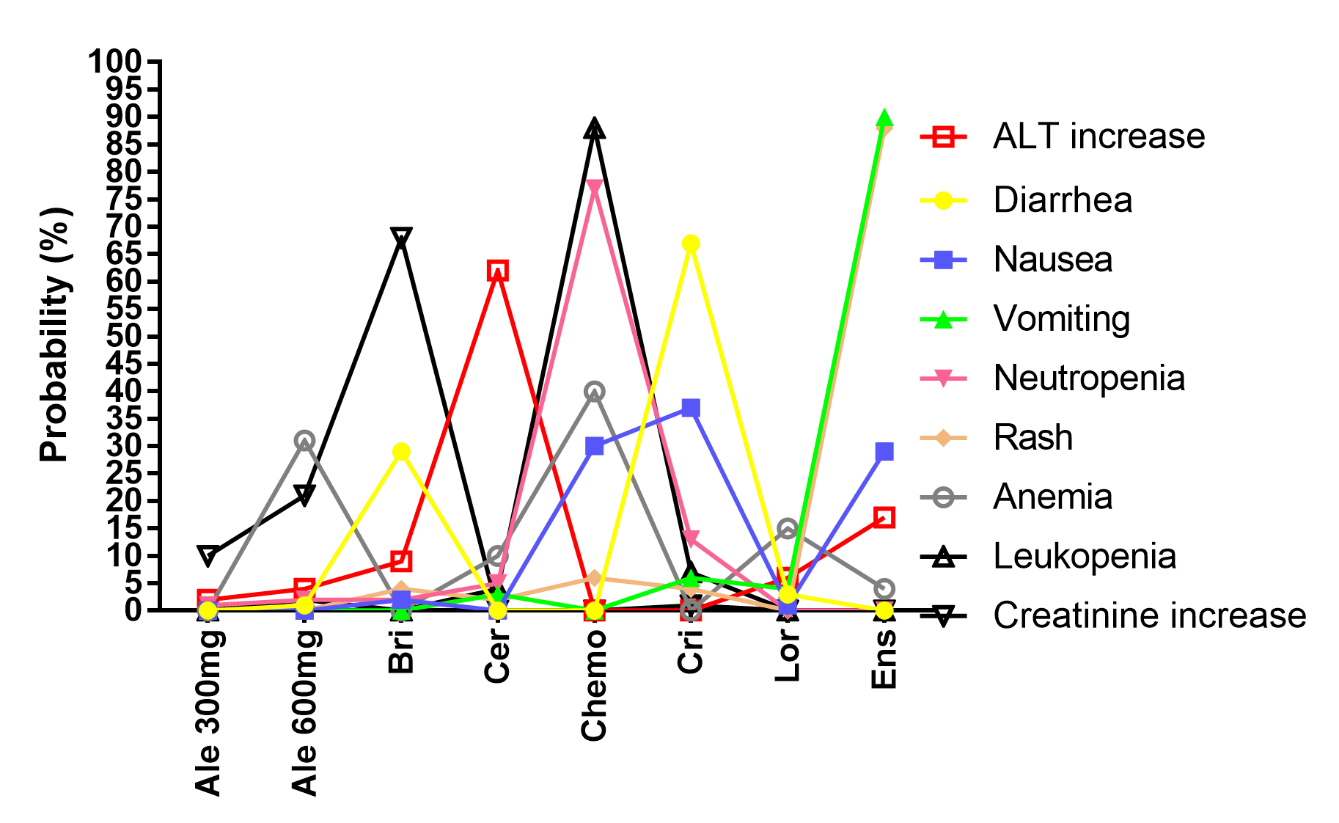


Figure S3 **Relative toxicity of treatments on nine commonly reported specific adverse events for ALK-TKIs(Ranking curves indicating the probability of each comparable treatment being ranked first on each specific adverse event).** Ale 300mg, alectinib 300mg; Ale 600mg, alectinib 600mg; Bri, brigatinib; Cer, ceritinib; Chemo, chemotherapy; Cri, crizotinib; Ens, ensartini; Lor, lorlatinib.


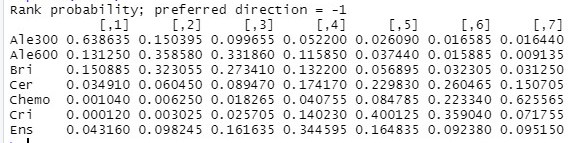


**Figure S4** **Bayesian ranking profiles of comparable treatments on PFS for patients with the baseline CNS metastases**. Ale300, alectinib 300mg; Ale600, alectinib 600mg; Bri, brigatinib; Cer, ceritinib; Chemo, chemotherapy; Cri, crizotinib; Ens, ensartinib; CNS, central nervous system.


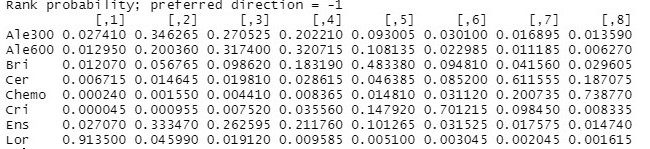


**Figure S5** **Bayesian ranking profiles of comparable treatments on PFS for patients without baseline CNS metastases**. Ale300, alectinib 300mg; Ale600, alectinib 600mg; Bri, brigatinib; Cer, ceritinib; Chemo, chemotherapy; Cri, crizotinib; Ens, ensartinib; Lor, lorlatinib.; CNS, central nervous system.


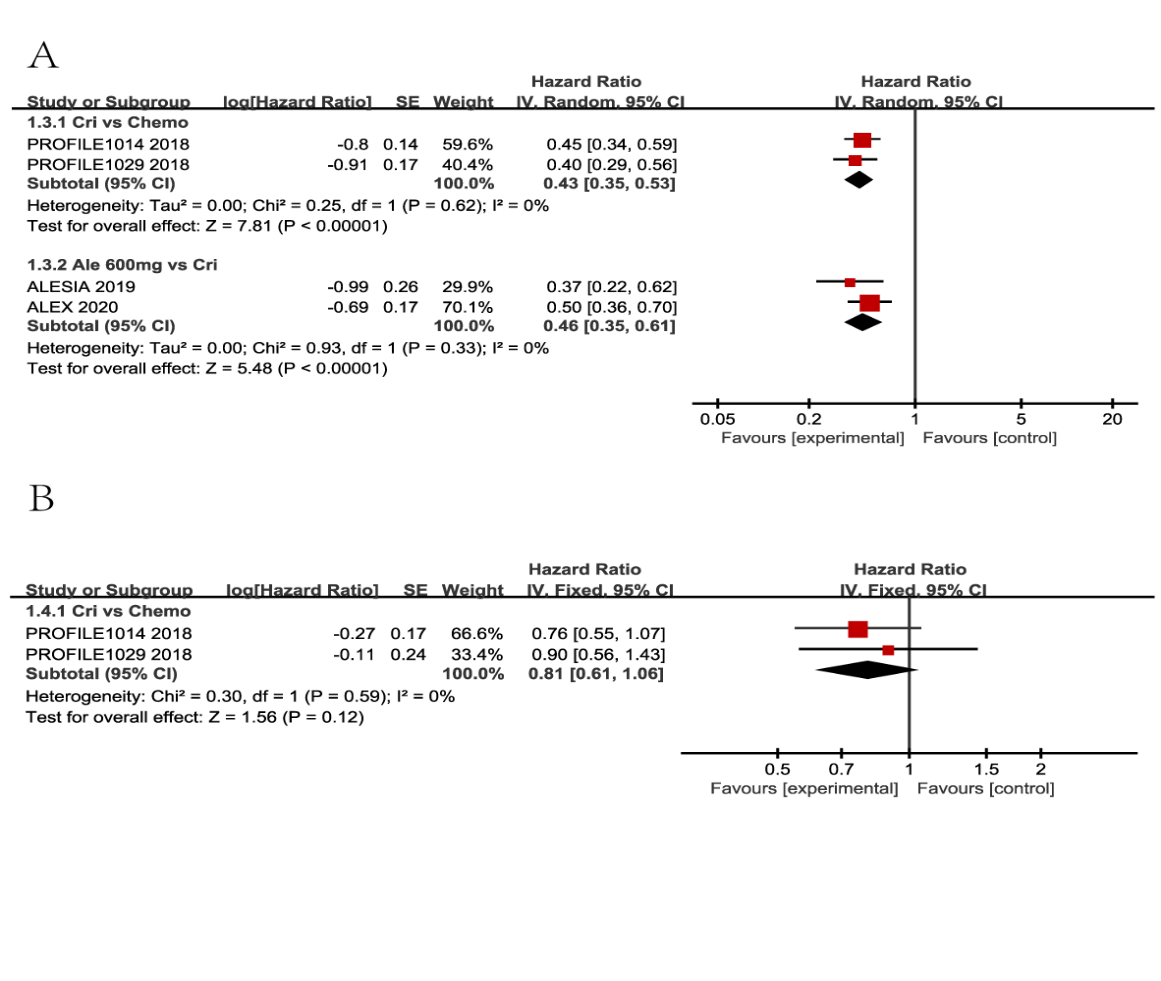


**Figure S6** **Forest plots of head-to-head comparisons for PFS and OS.** (A) head-to-head comparisons on PFS. (B) head-to-head comparisons on OS. Ale 600mg, alectinib 600mg; Chemo, chemotherapy; Cri, crizotinib; PFS, progression free survival; OS, overall survival.


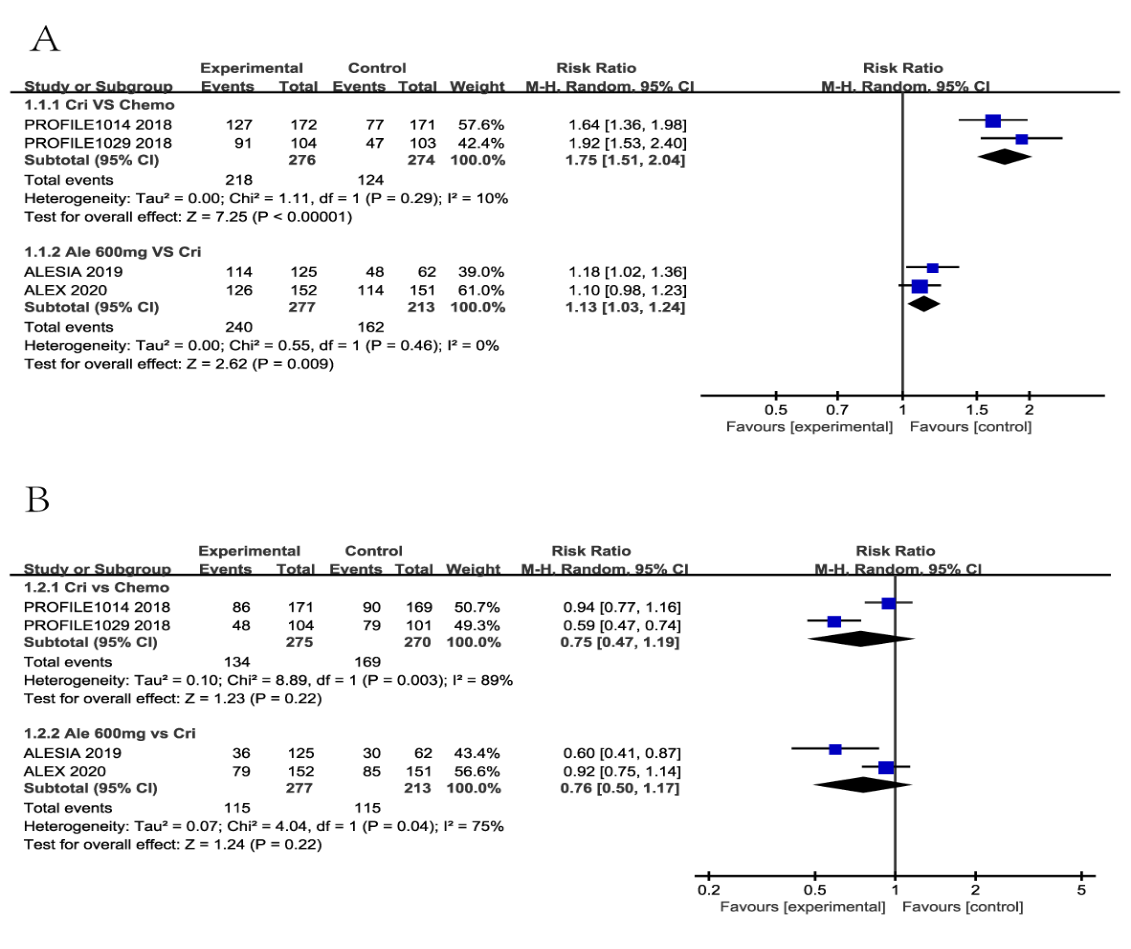


**Figure S7** **Forest plots of head-to-head comparisons for ORR and Grade≥3 AEs.** (A) head-to-head comparisons on ORR. (B) head-to-head comparisons on Grade≥3 AEs. Ale 600mg, alectinib 600mg; Chemo, chemotherapy; Cri, crizotinib; ORR, objective response rate; Grade≥3 AEs, adverse events of grade 3 or higher.


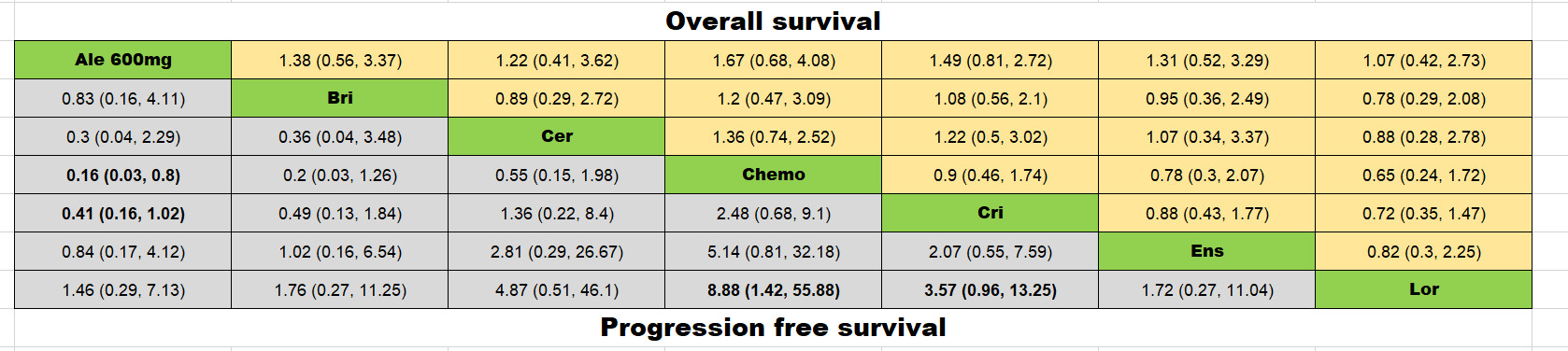


**Figure S8** Pooled estimates of the sensitivity analysis excluding the J-ALEX study: pooled hazard ratios (95% credible intervals) for overall survival (upper triangle) and progression-free survival (lower triangle).Ale 600mg, alectinib 600mg; Bri, brigatinib; Cer, ceritinib; Chemo, chemotherapy; Cri, crizotinib; Ens, ensartinib; Lor, lorlatinib.


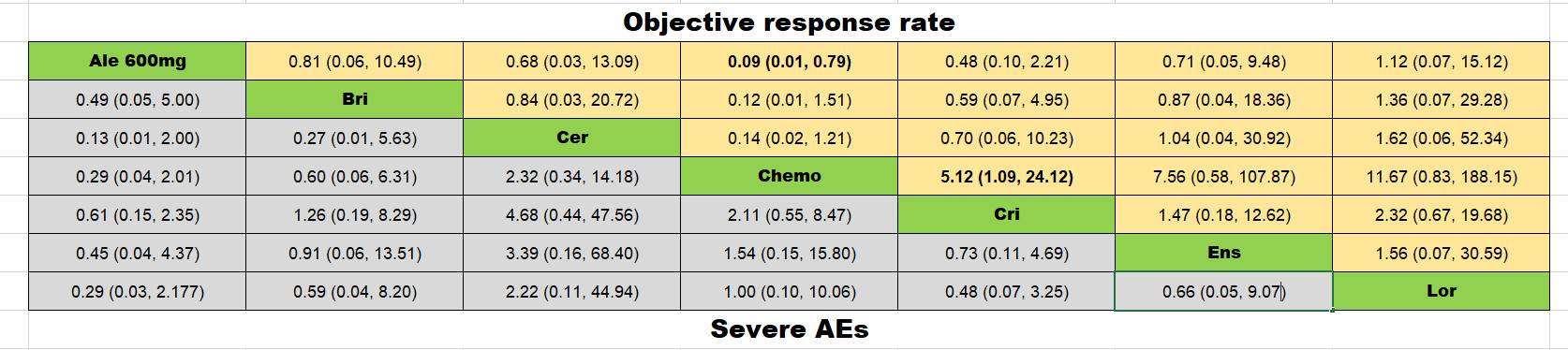


**Figure S9** Pooled estimates of the sensitivity analysis excluding the J-ALEX study: pooled odds ratios (95% credible intervals) for objective response rate (upper triangle) and grade ≥3 adverse events (lower triangle).Ale 600mg, alectinib 600mg; Bri, brigatinib; Cer, ceritinib; Chemo, chemotherapy; Cri, crizotinib; Ens, ensartinib; Lor, lorlatinib.
